# Supplementary material for: Predicting visual function by interpreting a neuronal wiring diagram
Source: Nature. 2024 Oct 2;634(8032):113–23. doi: 10.1038/s41586-024-07953-5 (PMC11446822; doi:10.1038/s41586-024-07953-5)
Supplement: Supplementary file 6 — CRF and ERF predictions for individual TmY4 and TmY9 cells. Analogous to Supplementary Data 3, but for TmY target types. Shown are the top four monosynaptic pathways, the strongest pathway passing through each of the top ten intermediary types (ranking from Extended Data Fig. 7), and the trisynaptic pathway Tm1–TmY–Dm3–TmY (see the section entitled Prediction of spatial normalization). [file 41586_2024_7953_MOESM6_ESM.zip › DataS4/TmY9q/720575940624081131.pdf]

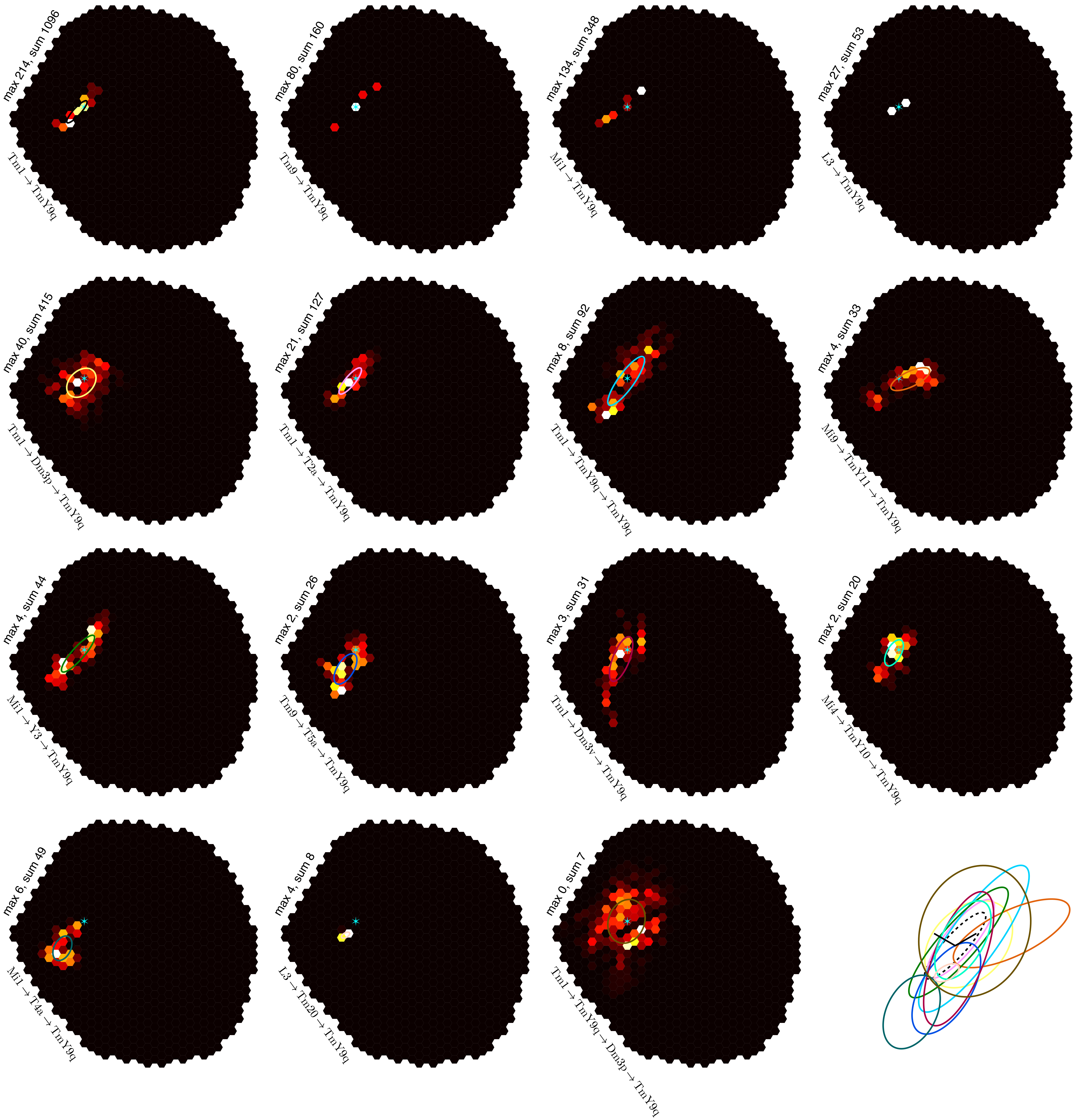
max 214, sum 1096
Tm1 → TmY9q
max 80, sum 160
Tm9 → TmY9q
max 134, sum 348
Mi1 → TmY9q
max 27, sum 53
L3 → TmY9q
max 40, sum 415
Tm1 → Dm3p → TmY9q
max 21, sum 127
Tm1 → T2a → TmY9q
max 8, sum 92
Tm1 → TmY9q → TmY9q
max 4, sum 33
Mi9 → TmY11 → TmY9q
max 4, sum 44
Mi1 → Y3 → TmY9q
max 2, sum 26
Tm9 → T5a → TmY9q
max 3, sum 31
Tm1 → Dm3v → TmY9q
max 2, sum 20
Mi4 → TmY10 → TmY9q
max 6, sum 49
Mi1 → T4a → TmY9q
max 4, sum 8
L3 → Tm20 → TmY9q
max 0, sum 7
Tm1 → TmY9q → Dm3p → TmY9q
